# Supplementary material for: Development and Characterization of a New TILLING Population for Forward and Reverse Genetics in Barley (Hordeum vulgare L.)
Source: Plants (Basel). 2024 Sep 5;13(17):2490. doi: 10.3390/plants13172490 (PMC11397183; doi:10.3390/plants13172490)

A

PW1:

AATCAAGGCTAGAAAGACTGGAACA|349bp|A|929bp|TCCTTTGATTACCTTAGCGAGTTCTT

PW2:

AATCAAGGCTAGAAAGACTGGAACA|349bp|C|929bp|TCCTTTGATTACCTTAGCGAGTTCTT

B

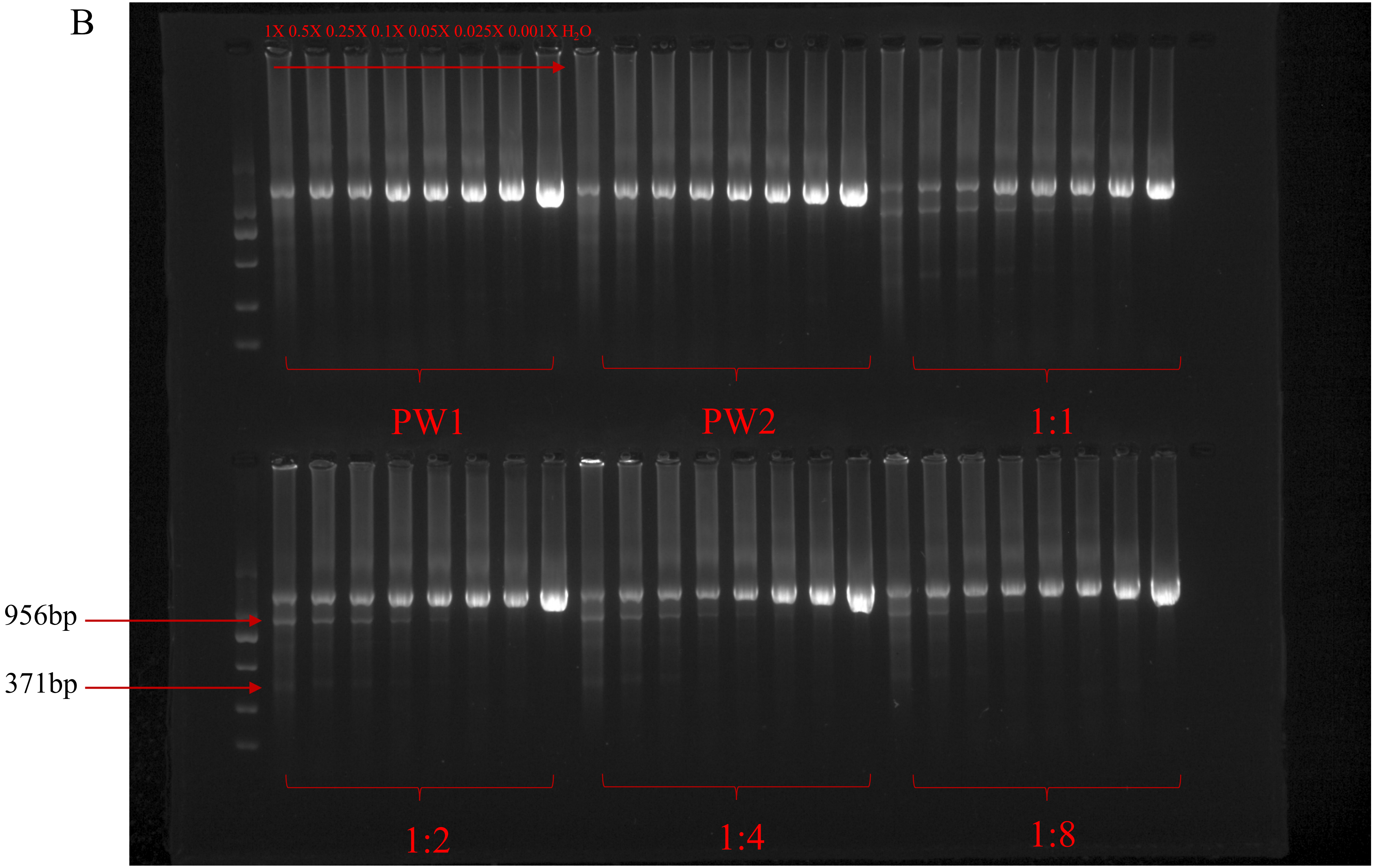

Supplement: Supplementary file 1 [file plants-13-02490-s001.zip › plants-3175362-supplementary.pdf]
